# One step PCR for detection of Staphylococcus aureus specific sequence gene and mecA gene in Northwestern Nigerian hospitals

**DOI:** 10.1186/2047-2994-4-S1-P196

**Published:** 2015-06-16

**Authors:** AS Kumurya

**Affiliations:** 1 Medical Laboratory Science, Bayero University Kano, Kano, Nigeria

## Introduction

Methicillin – resistant *Staphylococcus aureus* (MRSA) has been noted as one of the main pathogen of public health importance. Detection of the *mec* A gene by polymerase chain reaction (PCR) is the gold standard for identifying methicillin-resistant *Staphylococcus aureus*.

## Objectives

In order to accelerate the procedure of identification in clinical microbiology laboratories, it is very important to havea simple and rapid method for DNA extraction. In this work, a one step PCR assay for the detection of clinically relevant antibiotic resistance gene (*mec* A gene) harbored by some *Staphylococcus aureus* isolates and for the simultaneousidentification of such isolates at the species level has been described.

## Methods

In this work, a rapid method for bacterial DNA extraction directlyfrom a single colony that gave quality DNA for PCR in as littleas 15 minutes was used. PCR was used to amplify both the *Staphylococcus aureus* specific sequence gene and *mec* A gene of 100 isolates in Northwestern Nigeria with the amplicon size of 107 and 532 bp respectively. The performance and robustness of the assay was evaluated with a control strain of methicillin susceptible *Staphylococcus aureus*(MSSA).*-* ATCC 25923.

## Results

All the isolates (n=100) expressed *Staphylococcus aureus* specific sequence gene in their PCR products. Only 5 isolates (5.0%) were confirmed as MRSA based on the detection of *mec* A gene. This protocol yielded good-quality target DNA for PCRamplification. Amplifications using that DNA gave rise to goodquantities of the expected PCR fragments.

## Conclusion

This assay offers a rapid, simple, feasible, specific, sensitive, and accurate identification of MRSA clinical isolates and could be systematically applied as a diagnostic test in clinical microbiology laboratories, facilitating the design and use of antibiotic therapy. Hence, considering that it represents a cost-effective method and helping treatment to be initiated withoutdelay.

## Disclosure of interest

None declared.

